# Peak Detection Algorithm for Vital Sign Detection Using Doppler Radar Sensors †

**DOI:** 10.3390/s19071575

**Published:** 2019-04-01

**Authors:** Ju-Yeon Kim, Jae-Hyun Park, Se-Young Jang, Jong-Ryul Yang

**Affiliations:** 1 Department of Electronic Engineering, Yeungnam University, Gyeongsan, Gyeongbuk-do 38541, Korea; kjy102713@ynu.ac.kr (J.-Y.K.); bravopark@ynu.ac.kr (J.-H.P.); 2 Department of Life Science, Yeungnam University, Gyeongsan, Gyeongbuk-do 38541, Korea; dale47@ynu.ac.kr

**Keywords:** continuous-wave Doppler radar, electrocardiography, heart rate variability analysis, heartbeat, SDNN, RMSSD, autonomic nervous system, vital signs, peak detection

## Abstract

An accurate method for detecting vital signs obtained from a Doppler radar sensor is proposed. A Doppler radar sensor can remotely obtain vital signs such as heartbeat and respiration rate, but the vital signs obtained by using the sensor do not show clear peaks like in electrocardiography (ECG) because of the operating characteristics of the radar. The proposed peak detection algorithm extracts the vital signs from the raw data. The algorithm shows the mean accuracy of 96.78% compared to the peak count from the reference ECG sensor and a processing time approximately two times faster than the gradient-based algorithm. To verify whether heart rate variability (HRV) analysis similar to that with an ECG sensor is possible for a radar sensor when applying the proposed method, the continuous parameter variations of the HRV in the time domain are analyzed using data processed with the proposed peak detection algorithm. Experimental results with six subjects show that the proposed method can obtain the heart rate with high accuracy but cannot obtain the information for an HRV analysis because the proposed method cannot overcome the characteristics of the radar sensor itself.

## 1. Introduction

Drowsiness has become a major social issue due to the increase in nighttime personal and social activities. For example, drowsiness is a major cause of large-scale traffic accidents as well as a decrease in work performance and other daytime activities during the daytime [1]. In particular, various technical methods have been studied to prevent drowsy driving because it threatens an unspecified number of lives [2].

Drowsiness is a physiological, continuous response due to the action of the autonomic nervous system [3]. There is a time difference between the onset of cognitive drowsiness (confirmed by external features) and the physiological drowsiness (judged by biological signals). Thus, detection of physiological drowsiness can allow time to cope with the problems posed by later-onset cognitive drowsiness [4]. Analyzing the characteristics of the physiological drowsiness that change continuously in stages can predict the occurrence of cognitive drowsiness [5]. Previous technologies used to detect the physiological drowsiness have generally been based on electrocardiography (ECG) and electroencephalography (EEG) signals [6,7,8]. Sensing technologies based on EEG signals are used to distinguish the sleep stage because signals in the alpha band (8−13 Hz) are related to sleep stage 1, which indicates drowsiness [9,10]. However, the previous study reported that drowsiness recognition by EEG signals was 85% reliable, which was lower than that of ECG signals, which are 97.5% reliable [11]. ECG signals are used in many types of research to identify the relationship between the heartbeat and physical activities or diseases because ECGs can show the changes and irregularities in the heartbeat caused by the autonomic nervous system [12]. Heart rate variability (HRV) analysis, which shows irregularities in the heartbeat obtained by ECG signals, is a widely used drowsiness indicator for those studies [13]. The ECG signals in the low frequency (LF, 0.04–0.15 Hz) represent the activation degree of the sympathetic nervous system, whereas signals in the high frequency (HF, 0.15–0.4 Hz) bands represent the activation degree of the parasympathetic nervous system. Compared to the power level in the awakening state, the drowsiness state decreases power in the LF band and increases power in the HF band. The ECG signals in the time domain can also describe the drowsiness state using the standard deviation of normal-to-normal peaks (SDNN), which is the standard deviation of the time interval between R-peaks in the ECG signals. This is because the SDNN is known to be associated with the signals in the alpha band of the EEG [8]. There is, however, a fundamental limit to drowsiness recognition using ECG and EEG signals because electrodes must remain connected to the human body during the measurement process and instability of the electrodes’ contacts may distort the measurement results [14]. Therefore, ECG and EEG have limitations for use in industrial applications such as a driver drowsiness monitoring system.

A radar sensor based on the Doppler effect can detect the vital signs by determining the difference between transmitted and received electromagnetic waves [15]. These vital signs show the microscopic movement of the internal organs of the human body in response to the heartbeat and respiration. The Doppler radar sensor might use in the HRV analysis like ECG when the signal waveform from the radar is obtained in a similar waveform as ECG. However, because of the operating principle of the radar sensor, it is difficult to accurately detect the R-peak position of the ECG signal, which is used to analyze the specificity, irregularity, and change of the heartbeat in time domain. Signal processing methods based on the fast Fourier transform and the parameter optimization using the nonlinear algorithm have conventionally been used to detect the vital signs in the radar sensor instead of the exact peak detection used in ECG [16,17]. The conventional methods of EEG and ECG have limitations that prevent the detection of vital signs in real-time because a large number of samples are required to obtain a level of data accuracy that can clearly distinguish vital signs located in the frequency range near 1 Hz. The slow signal processing speed and the long data acquisition time of the conventional approach also mean that these methods cannot be used to process signals in real-time obtained from the radar sensor for specificity and nonuniformity of the heartbeat over time.

In this paper, a peak detection algorithm to detect vital signs is proposed as a method to accurately detect periodic characteristics after considering and accounting for the specificity and operating principle of the radar sensor. Measurement results using the radar sensor in conjunction with the peak detection algorithm show that the proposed method can accurately detect the number of peaks of the heartbeat signal from the raw data at a faster processing time than the conventional method. The proposed method can also obtain a high degree of accuracy that is comparable to the ECG. The output signals of the radar sensor processed by the peak detection algorithm are analyzed by applying an HRV analysis used in the ECG approach and compared to the results obtained from the reference ECG sensor for verifying the possibility of drowsiness prediction. The measurement results of six subjects show that the radar sensor with the proposed algorithm cannot be applied for HRV analysis and drowsiness prediction even though the algorithm has high accuracy for measuring the number of the peaks count depending on the time variation. In Section 2, the differences between the raw data from the Doppler radar sensor and the ECG signals are described and the peak detection algorithm is shown to indicate that the radar sensor accurately detected the vital signs. Next, Section 3 presents the experimental setup, including the implemented Doppler radar sensor and the measurement process, to demonstrate the performance improvement offered by the peak detection algorithm as well as the possibility of the drowsiness prediction from the HRV analysis. The results and discussion of the measurements and analysis are provided in Section 4 and the conclusion is shown in Section 5. 

## 2. Theory and Methods

### 2.1. Differences between Radar Sensor Output and ECG Signals

**Figure 1:**
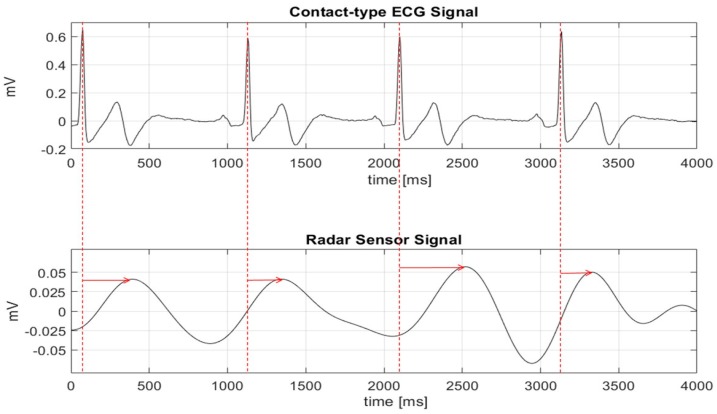
Cardiac-related radar sensor signal and ECG signal comparison in the time domain.

The heartbeat is regulated by the cardiac conduction system in the heart, which is itself related to the autonomic nervous system [18]. One cycle of the heartbeat consists of atrial and ventricular contractions, which process in the following order: (1) The atrial contraction is caused by an electrical action potential produced by the sinoatrial node, which is one of the nodal tissues that regulate the heartbeat, through myocardial cells. (2) As the atrial contraction occurs, the current generated by the action potential is transferred to the atrioventricular node. (3) After the atrial contraction is complete, the signals from the atrioventricular node are transmitted to the bundle of the His fibers and the Purkinje fibers, which causes a ventricular contraction in both ventricles. In ECG, the atrial contraction is represented by the P-wave, and the ventricular contraction is represented by a series of QRS complex waveforms. Of particular note, the R-wave in the QRS waveform shows the maximum magnitude attainable in a single cycle of the waveform. Compared with the ECG, which represents the heartbeat as electrical signals, the radar sensor shows the heartbeat by detecting the periodic phase shift between the transmitted and the received signals generated by the movement of the thoracic cavity [19]. In addition to the movement of the external thoracic cavity by respiration, micro-vibrations of the chest wall (which occurs as the chest cavity due to the ventricular contraction) can also be observed using the radar sensor. Based on the operating principle of the radar sensor, the vital signals obtained using the sensor show mixed signals comprised of both the fine heartbeat signals produced by the atrium and ventricular motions and the large respiration signals produced by the movement of the chest cavity. In particular, the atrium and ventricular motions are not distinguished from one other in the sensor, and the heartbeat signals in the radar sensor don’t show clear peaks like the ECG. As shown in Figure 1, the heartbeat waveforms in the time domain measured by the radar and the ECG sensors don’t exhibit identical peak positions. 

**Figure 2:**
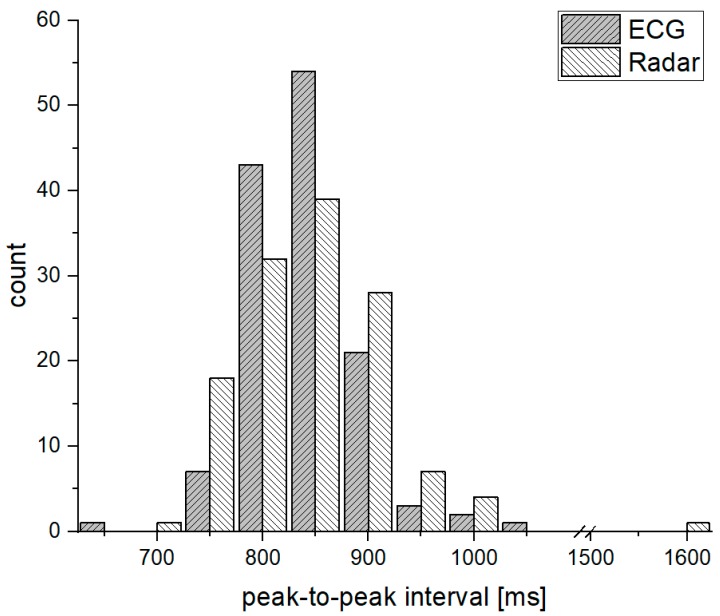
Histogram of the peak-to-peak interval for the measured ECG and radar signals.

Figure 1 also shows that although the exact peak location cannot be found, the number of peaks at a given time is the same between the radar and the ECG sensors. Figure 2 shows the histogram of the peak-to-peak intervals measured by the ECG and radar sensors at the same time. The phase delay in the signal conditioning block can generally produce a constant movement of the peak positions in the time domain. However, the differences in the peak positions between the signals obtained from the ECG and radar sensors are not caused by the phase delay. The peak intervals obtained from the radar sensor are distributed over a wider range than those obtained from the ECG sensor at the same number of peaks. Therefore, analysis of the heartbeat signals obtained from the radar sensor should use the number of peaks in a defined period of time, and it is necessary to accurately count the number of peaks contained in the raw data. Additionally, the peak size and the time interval between the peaks in the time domain can be used within the data obtained by the radar sensor.

### 2.2. Proposed Peak Detection Algorithm

**Figure 3:**
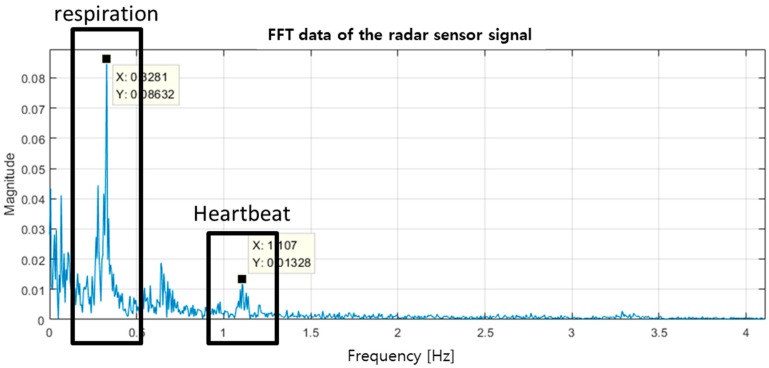
Respiration and heartbeat signals in the frequency domain measured using the radar sensor.

Two conventional methods are used to detect the peak value of the heartbeat signal in the time-domain obtained from the ECG: the adaptive moving maximum value detection, which finds the highest value in the data collected within a certain interval, and the gradient-based method, which locates the point at which the slope becomes zero at the maximum value [20,21]. These conventional methods function well for ECG signals because the peak and magnitude of the R-wave are clearly distinguished from the others. However, these conventional methods cannot be used in the radar sensor due to the increased error rate. This is caused by various movements inside and outside the body, which create several local maxima and minima between the peaks. The preceding process of eliminating the noise signals from the body movement and the power supply must be performed to extract only the vital signs from the radar sensor. The 60 Hz noise signal from the wired power supply can be removed by using a band rejection filter, but the noise signals due to the macro motion by the body cannot be eliminated by a general filtering method because it has a frequency similar to the vital signals. An experimental environment that minimizes the subject’s macro motions is mandatory to increase the radar sensor’s accuracy in acquiring the signal. The noise-minimized vital signals cluster in the specific frequency bands due to the homeostasis of the human body. The persons in the stable arousal state without respiratory and cardiac disease generally respire 15 to 20 times per minute and have a heartbeat of 60 to 90 beats per minute [22]. Figure 3 presents the measurement data in the frequency domain using the radar sensor. It separates the respiration and heartbeat signals into two separate ranges: the 0.2 to 0.33 Hz range, and the 1 to 1.5 Hz range, respectively. The respiration and heartbeat rates increase or decrease from the normal state when activities such as exercise and sleep activate the autonomic nervous system. The frequency band of the heartbeat in the drowsy state varies from 0.8 to 2 Hz based on the previous analysis in which the minimum beat-per-minute (BPM) was found to be as low as 40 BPM based on the sleeping heart rate [23]. Respiration signals are filtered out using a high-pass filter with a cut-off frequency of 0.5 Hz because the heartbeat signals are only used in the HRV analysis for drowsiness-state detection.

**Figure 4:**
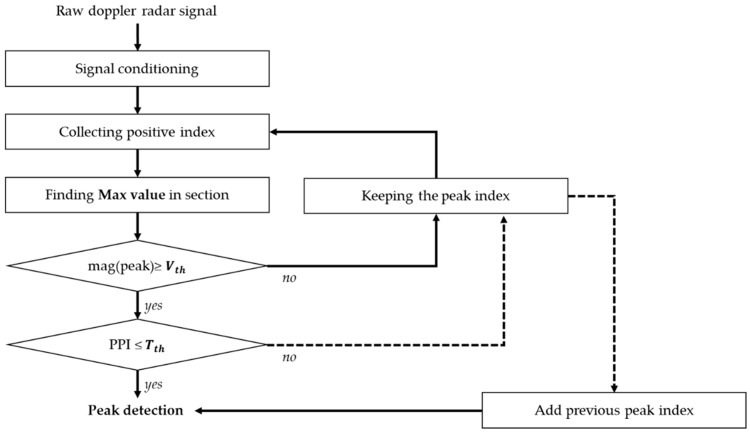
Overall flowchart of the proposed peak detection algorithm.

The heartbeat signal in the time domain is presented by a waveform with the baseline voltage of 0 V because the DC offset is removed using the high-pass filter. The peaks in the waveform are found by the zero-crossing method, which recognizes the number of peaks by assuming that at least one peak is located between waveforms that change in opposite polarity. The zero-crossing method can show more peaks than the actual value due to the remaining noise signals changing near the 0 V baseline and the harmonic signals from respiration in a similar frequency band. Because the amplitudes of the noise signals are distributed over a wide frequency band and are smaller than those of the heartbeat signals after several filtering processes, the accuracy of the peak detection in the proposed algorithm can be improved by using the threshold as the baseline voltage. The threshold voltage in the algorithm is defined as half the value of the averaged RMS (root-mean-square) voltage of the magnitudes of all the peaks obtained from the zero-crossing method as:

(1) $$V_{th}=\;\frac{1}{2n}\overset{n}{\underset{i=1}{\sum }}\frac{\left|V_{mag}\left(i\right)\right|}{\sqrt{2}},$$

where *V_th_* is the threshold voltage, *n* is the overall number of peaks from the zero-crossing method in a certain period that is the same as the window size in the HRV analysis, and *V_mag_*(*i*) is the magnitude in each peak, *i*. When the peak interval exceeds the reference value as a result of erroneous erases by the threshold voltage level, the previous peak detected by using the zero-crossing method is brought into the number of peaks. The reference value is defined from the frequency band of the heartbeat and shown in *T_th_* of Figure 4, which gives a flowchart of the proposed peak detection algorithm. The mean heart rate (mHR) of the HRV analysis using the proposed algorithm can be expressed as:

(2) $$mHR=\frac{n_{d}}{T}\times 60,$$

where *n_d_* is the number of the peaks detected using the proposed algorithm shown in Figure 4 and *T* is the unit time for data analysis, as well as the time length for counting the number of peaks. *T* can be described as the data index corresponding to the time position of the peak. 

### 2.3. Drowsiness Prediction Based HRV Analysis

It is generally considered that the number of peaks obtained from the radar sensor can be analyzed similarly to the R-peak of the ECG signal. However, the same analysis method cannot be used in the radar sensor because its peak-to-peak intervals in the radar sensor are not exactly the same as the R-peak intervals in the ECG. It is necessary to check whether the peak intervals obtained from the radar sensor with the proposed algorithm, which can accurately detect the number of peaks in the unit time, can apply to the HRV analysis (which is used in the ECG approach to diagnose disease presence or to observe continuous changes inside the body) and wake–drowsiness state detection [24]. 

The HRV analysis is performed through a general ECG signal and consists of both time domain analysis and frequency domain analysis. The frequency domain analysis requires a raw ECG signal including T and U waves as well as PQRS waves. Therefore, the frequency domain analysis has limitations because it requires either high sensor specifications or complex signal processing. On the other hand, the time domain analysis (which is being actively studied) focuses on the variability of the R-peak (which is the heartbeat) and deals with the number of R-peaks and its time interval information. As a person gets closer to the drowsiness state, activation of the parasympathetic nervous system decreases the heart rate. However, there is a variation in each individual’s heartbeat and it is therefore difficult to judge the drowsiness state heart rate alone—additional parameters related the autonomic nervous system activity are required. Table 1 shows the parameters of the definition and the characteristics of the SDNN and Root Mean Square of Successive Deviation (RMSSD). These parameters are well-known to show the autonomic nervous system effects of various time domain parameters. SDNN especially is related to the overall power spectrum, Ultra Low Frequency (ULF), and RMSSD is related to High Frequency (HF) in the frequency domain analysis parameter. These are both related to periodic biorhythm and autonomic nervous system activity. These parameters can be applied on the radar sensor data detected by the proposed algorithm for drowsiness-state prediction if the statistical data using the peak-to-peak intervals of the radar sensor do not differ significantly from the data using the ECG sensor. The SDNN and RMSSD can be individually expressed as:

(3) $$SDNN=\sqrt{\frac{\sum_{i=1}^{n}\left({\left(x_{i}-m\right)}^{2}\right)}{n-1}},$$

(4) $$RMSSD=\sqrt{\frac{\sum_{i=2}^{n}\left({\left(x_{i}-x_{i-1}\right)}^{2}\right)}{n-2}},$$

where *x_i_* is the *i*-th peak-to-peak interval value, *n* is the number of *x_i_*, and *m* is the average of *x_i_*. The radar sensor signal requires a new interpretation of time-interval-related information because it differs from the ECG signal. Because the heart rate can be accurately detected by the peak counting algorithm even for the radar sensor that is difficult to find the exact peak position, the heart rate-based statistical analysis parameter is required in the radar sensor for the HRV analysis and drowsiness-state prediction. The standard deviation of the rate-to-rate (SDRR), which shows the degree of the variability of the mHR, is defined as:

(5) $$SDRR=\sqrt{\frac{\sum_{i=1}^{k}\left({\left(mHR_{i}-mHR_{avr}\right)}^{2}\right)}{k-1}},$$

where *mHR_i_* is the *i*-th mean heart rate, *mHR_avr_* is the average of *mHR_i_*, and *k* is the number of *mHR_i_*.

The length of the analysis window is adjusted to check whether the statistical parameters which are the mHR, SDRR, SDNN, and RMSSD detected from the radar sensor signal have the same tendencies as the parameters detected from the ECG signal.

A window of 300 s is used in the analysis because the previous study reported the significant meaning to analysis windows of 5 min [25]. The analysis also examines the 60 s window to see whether the judgment is faster. The data of the mHR and SDRR in the window are continuously updated in every one second with a sampling rate of 1000 samples per second. The mHR and SDRR in each window are obtained from the continuously accumulated data. The SDNN and RMSSD are obtained and analyzed by using non-overlapping windows of 60 and 300 s.

## 3. Experiments

### 3.1. Subjects

The subjects were two men and four women in their 20s who did not have cardiac or sleep-related diseases, as shown in Table 2. The subjects were required not to consume caffeine, alcohol, or nicotine for more than 12 h before the measurements to minimize autonomic changes due to external influences. During the measurement, the observer records the drowsiness state, and after the measurement asks the subject to describe their state of self-recognition. The observer records at 20 s intervals whether the eyes are open or closed, the eyes’ closing time, and whether eyelid movement is sustained when the eyes are closed. A drowsiness state is defined as the time when eyes are closed for more than 20 s and eye movements don’t occur in the eyelids for more than 20 s [9,26,27]. The determination of drowsiness also includes the status and the time at which the subjects replied that they were in a state of drowsiness after the experiment. A cognitive drowsiness state in our study is defined as sleep stage 1 as determined in the previous study, or the state in which the observer can check whether the subject’s drowsiness starts [9].

### 3.2. Radar Sensor and Measurement Environment

**Figure 5:**
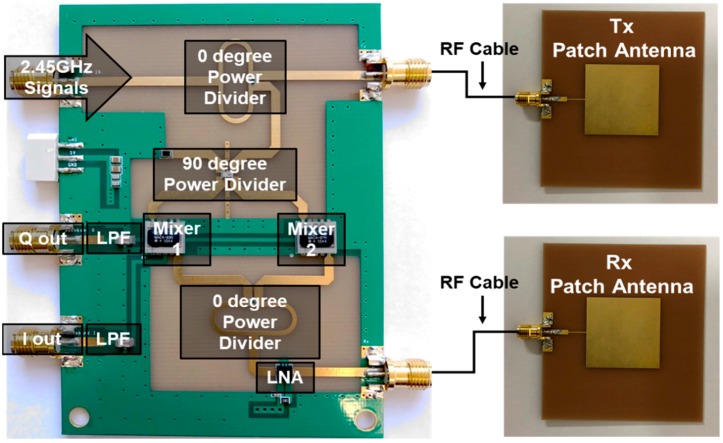
A 2.45-GHz quadrature continuous-wave Doppler radar sensor for vital signs detection.

A quadrature continuous-wave (CW) Doppler radar sensor module and two antennas were implemented on an FR4 printed circuit board (PCB) with a thickness of 1 mm, as shown in Figure 5. 2.45 GHz signals are generated on the N5183B signal generator (Keysight, Santa Rosa, CA, USA) with an output power of 10 dBm. The signals are divided at the Wilkinson power divider, which has two in-phase outputs, into the reference signal and the transmitted signal. The transmitted signal is radiated toward the subject through the transmitting antenna with a directivity of 5.9 dBi, and the signal reflected by the subject is received at the receiving antenna which has the same characteristics as the transmitting antenna. The reference signal passes through a power divider with a 90° phase difference at the outputs to produce quadrature signals. The received signal is amplified at the low-noise amplifier (LNA) with a power gain of 13.68 dB and a noise figure of 5.3 dB, and then individually down-converted by two frequency conversion mixers with a conversion gain of −5.8 dB using the quadrature signals. The two signals converted into quadrature signals with a phase difference of 90° are classified into I and Q channels. The radar sensor using quadrature signals can reduce the effect of the “null point problem”, in which the signal amplitude seriously decreases depending on the position and distance of the subject [28]. The implemented radar sensor uses a higher channel output depending on the subject, without any demodulation techniques to recover the quadrature output signals because the signal-to-noise ratio (SNR) at the output is enough to distinguish the heartbeat signals. 

**Figure 6:**
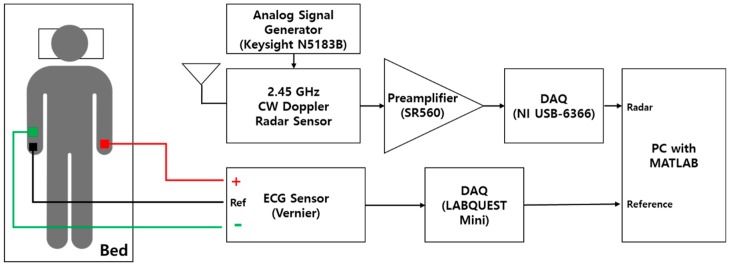
Measurement environment for vital signs monitoring.

Figure 6 shows the measurement environment for monitoring the subject’s vital signs using the radar sensor and a commercial contact-type ECG sensor. The low-noise preamplifier manufactured by Stanford Research Systems (Sunnyvale, CA, USA) amplifies the outputs with a voltage gain of 40 dB, and filters the noise signals with a passband frequency range from 0.03 to 3 Hz. The baseband signals traveling through the preamplifier are captured with a sampling rate of 1 k samples per second in the data acquisition (DAQ) board (National Instruments, Austin, TX, USA). The ECG sensor manufactured by Vernier Software & Technology (Beaverton, OR, USA) is used as the reference ECG sensor. ECG signals from three contact-type electrodes are recorded with a sampling rate of 200 samples per second. All the signals from both sensors are simultaneously sent to the PC and processed using MATLAB (MathWorks, Natick, MA, USA). During the measurement time of 20 mins, the subject was lying on a bed to minimize the noise signal created by the subject’s movement. The distance between the antennas and the subject was fixed to approximately 40 cm because of the limitation of the distance between the bed and the sensor support. Although the radar sensor does not obtain signals in the far-field condition, the sensor shows a sufficient SNR for vital signs detection as shown in Figure 3 [29].

## 4. Results and Discussion

**Figure 7:**
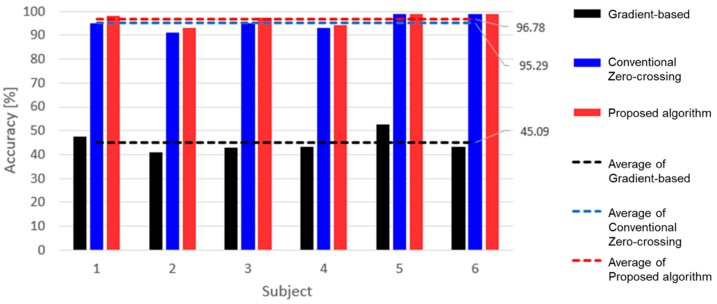
Peak detection accuracy of the gradient-based, the conventional zero-crossing, and the proposed peak detection algorithms.

The improvement by the proposed algorithm in the radar sensor can be expressed by using the accuracy of the mHR and computation speed. The computation speed of the peak detection algorithm is checked by the CPU profiler provided by MATLAB, and the detection accuracy can be expressed as:

(6) $$Accuracy\;=\frac{HR_{ECG}-\left|HR_{ECG}-HR_{Radar}\right|}{HR_{ECG}}\times 100\;\left(\%\right),$$

where *HR_ECG_* is the mHR obtained from the number of R-peaks in the ECG waveforms and *HR_Radar_* is the mHR processed with the peak detection algorithm from the raw data using the radar sensor. Figure 7 shows the average accuracy using the mHR obtained from each algorithm in the window size of 60 s. The gradient-based pattern recognition algorithm is used as a reference to assess the performance of the proposed algorithm [30]. The gradient-based algorithm is not suitable to use with the vital signs detection in the radar sensor because the algorithm detects local minima and maxima, which should not be counted to obtain the heart rate. The conventional zero-crossing algorithm identifies as peaks the point at which the slope becomes zero for a signal mixed with respiration and heartbeat signals. As shown in Figure 7, the accuracy of the proposed algorithm is improved from the conventional algorithm because the peaks which have less amplitude are removed by the threshold level of the proposed algorithm. The average accuracy of the proposed algorithm as compared to the gradient-based algorithm increased from a mean of 45.09% with a standard deviation of 3.92% to a mean of 96.78% with a standard deviation of 2.32%. The difference of the accuracy between conventional and proposed algorithms has a mean of 1.49% and standard deviation of 0.49%. The results in Figure 7 show that the proposed algorithm has the highest accuracy and the lowest standard deviation in the comparison of the three algorithms. For the subjects who showed a high accuracy of mHR using the proposed algorithm, the accuracies of mHR using the other algorithms were also higher than the other subjects. These results show that the raw data obtained by the radar sensor can easily distinguish the peaks of the heartbeat signal from the noise due to the high SNR.

**Figure 8:**
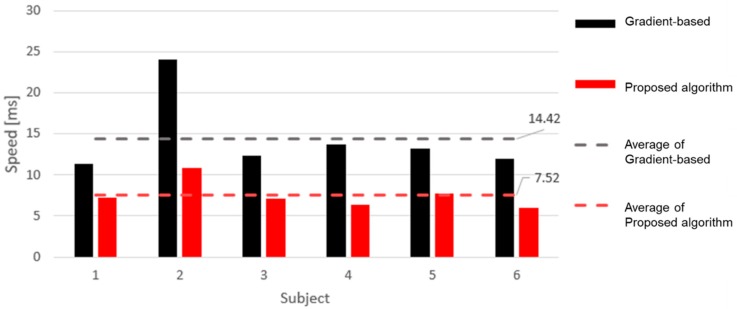
Calculation speed of the gradient-based detection algorithm and the proposed peak detection algorithm.

Figure 8 shows that the computation speed in the proposed algorithm was improved from 14.42 to 7.52 ms compared with the gradient-based algorithm. The twice faster speed is based on the fact that the proposed algorithm finds only the local maximum corresponding to the positive peak value in the time interval by considering the normal heartbeat period. As the window size increases, the specific processing time may increase in the procedure to store and read the peak index. However, the total processing time in the proposed algorithm does not increase significantly because the heart rate can be recognized without converting the original radar waveform. 

HRV was confirmed based on peaks detected from the radar sensor signal using both the proposed peak detection algorithm and the R-peaks output from the ECG signal. Output signals from the ECG and radar sensors were measured every 20 min for each subject and at least one drowsiness state in each subject was observed by the operator. Because the vital signs of a person may be changed by other factors except for the drowsiness, it is necessary to clearly distinguish the duration recognized as the drowsiness in order to confirm only the change of heartbeat signal due to drowsiness. The drowsiness period of each subject is defined as the term “event” when the drowsiness and awakening are repeated, and one event means that one period in the drowsiness state is observed. When a plurality of events occur in the same subject, the parameters in the awakening and drowsiness states are separately analyzed. The length of each event appeared irregularly in the same subject, and each subject observed one or two events during 20 min. 

**Figure 9:**
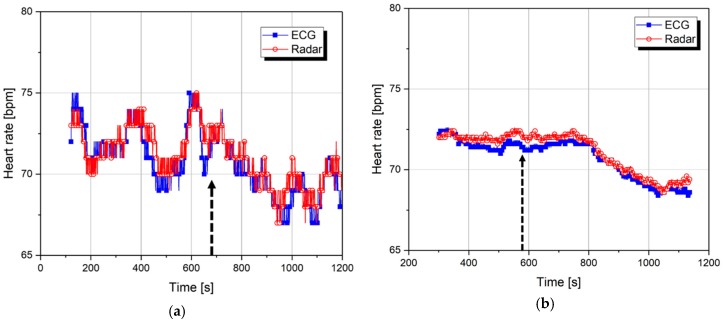
Variation of the heart rate for subject 5 in the wake–drowsiness states: (**a**) Using the window size of 60 s; (**b**) using the window size of 300 s.

**Figure 10:**
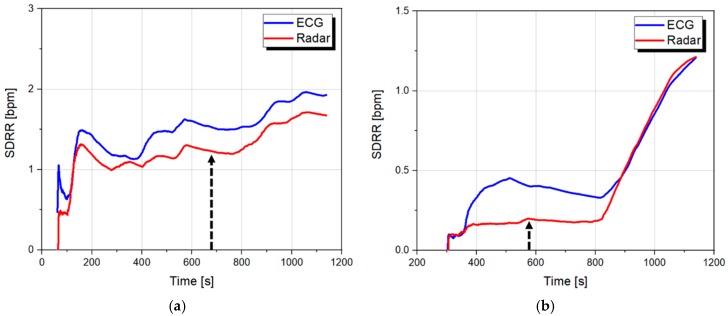
Variation of the SDRR for subject 5 in wake–drowsiness states: (**a**) Using the window size of 60 s; (**b**) using the window size of 300 s.

Figure 9 shows the changes in the heart rate obtained at each window size in 1-s intervals, and Figure 10 shows the SDRR obtained from the rate-to-rate interval in Figure 9 for clarifying changes in the heart rate. It is demonstrated that the heart rate obtained from the radar sensor agrees with the heart rate by the ECG sensor, and this is the same result as the previous study [31]. The previous studies, which analyzed ECG signals, showed that heart rate decreased due to activation of the parasympathetic nervous system when changing from an awake state to a light sleep state [32]. This study confirmed that the same tendency was observed in the indicated area for the parameters obtained from the radar sensor for the 60 s window, and was thus judged to be a physiological drowsiness state. The arrows shown in Figure 9 and Figure 10 indicate the starting point of the event, and the data shows that the heart rate is changed after the event occurs.

Because the gap between the time at the change of the heart rate and the time by the arrow is different in each window size, it can be understood that the heartbeat signal has a delay time in indicating the heart rate due to the window size. Therefore, the results in Figure 9 and Figure 10 show that drowsiness prediction is impossible with the variation of the heart rate measured from the number of peaks. The parameters for HRV analysis are shown in Table 3, Table 4, Table 5 and Table 6. The average parameters in the awakening state and the cognitive drowsiness state, which are distinguished by each event, were obtained with each window size. In Table 4, the ‘*’ indicates that the SDRR cannot be obtained because the drowsiness within the first window size cannot be clearly distinguished. The subjects with the heart rate accuracy over 95% compared to the ECG have a tendency toward a decreased heart rate and increased SDRR in the drowsiness state. The tendency shows that the HRV as a parameter of heart rate is related to the accuracy of the peak detection algorithm. As shown in Table 5 and Table 6, the SDNN and RMSSD obtained from the ECG increased in the drowsiness state, whereas the parameters from the radar sensor did not show a certain tendency. Also, the SDNN and RMSSD obtained from the radar sensor were significantly different from those obtained from the ECG sensor. These results were due to the intrinsic characteristics of the radar sensor, which cannot accurately detect peak positions. For the HRV analysis based on the SDNN and RMSSD, it is necessary to detect a precise peak position analyzed in addition to the algorithm for accurately detecting the number of peaks in a unit time. The experimental results in Table 3, Table 4, Table 5 and Table 6 show that the analysis results for the 60-s window and the 300-s window have similar tendencies. In general, it is difficult that the SDNN, which is a standard deviation of a given dataset, shows statistically quantified results using a short-length window compared to the RMSSD which shows the variation between neighboring data. However, the results show that a 60-second window can be used to monitor consecutive changes in awakening and drowsiness states for a short period of time.

## 5. Conclusions

A peak detection algorithm is proposed to solve the problem of finding the exact peak interval in the raw data produced by a radar sensor. The peak intervals located using the proposed algorithm are obtained with an mean accuracy of 96.78% when compared to the ECG signals, and the processing time is two times faster than that for the conventional algorithm. HRV analysis for predicting the drowsiness state is performed on six subjects to collect heartbeat data that is then processed with the proposed algorithm in the radar sensor. Window sizes of 60 and 300 s are applied to the HRV analysis during an overall measurement time of 20 min. The measurement results demonstrate that the heart rate and SDRR obtained from the processed data of the radar sensor describe the drowsiness on the basis of the changes in ECG signals and external features related to the drowsiness. The results also show that a new algorithm to accurately find both the peak positions and the number of peaks will be required for the radar sensor for drowsiness prediction based on the HRV analysis using SDNN and RMSSD.

## Figures and Tables

**Table 1:** Time-domain parameters of the HRV analysis.

Variable	Units	Definition	Characteristics
SDNN	ms	Standard deviation of all N-N intervals	It reflects all the long-term components and changes in the 24-h cycle rhythm.
RMSSD	ms	The square root of the mean of the sum of the squares of differences between adjacent N-N intervals	It is associated with short-term HRV changes and reflects changes in autonomic tone, independent of day and night changes.

**Table 2:** Survey results of subjects’ characteristics before the experiment.

Subjects	Age	Gender (M/F)	Drinking Condition	Smoking Condition	Caffeine Consumption Condition
1	23	F	None for the month	Non-smoker	None for 12 h
2	23	F	None for the week	Non-smoker	None for 12 h
3	25	M	None for the week	Non-smoker	None for 12 h
4	23	F	None for the week	Non-smoker	None for 16 h
5	22	F	None for the month	Non-smoker	None for 20 h
6	22	M	None for the week	Non-smoker	None for 20 h

**Table 3:** The average of the mHR in the wake–drowsiness states obtained from the analysis window.

Subject	Heart Rate by 60 s Window (BPM)				Heart Rate by 300 s Window (BPM)			
	ECG		Radar		ECG		Radar	
	Wake	Drowsiness	Wake	Drowsiness	Wake	Drowsiness	Wake	Drowsiness
1	67.00	66.10	67.00	65.19	68.60	65.73	68.40	65.93
2	81.12	82.35	74.44	74.02	81.32	81.48	76.10	74.60
3	84.09	73.85	81.17	73.29	84.72	75.67	81.31	75.26
4	86.23	84.08	76.83	79.60	87.00	83.65	79.00	80.04
5	71.87	69.05	72.04	69.57	71.69	69.94	72.02	70.24
6	75.32	63.63	75.41	63.85	73.80	64.79	73.20	64.87

**Table 4:** The average of SDRR in the wake–drowsiness states obtained from the analysis window.

Subject	SDRR by 60 s Window (BPM)				SDRR by 300 s Window (BPM)			
	ECG		Radar		ECG		Radar	
	Wake	Drowsiness	Wake	Drowsiness	Wake	Drowsiness	Wake	Drowsiness
1	*	1.57	*	1.69	*	1.03	*	0.88
2	2.00	1.96	2.79	3.19	0.55	0.71	0.83	1.31
3	1.74	4.72	2.51	3.76	0.42	3.45	0.48	2.24
4	0.68	2.29	1.08	2.68	*	1.28	*	0.89
5	1.27	1.74	1.05	1.49	0.32	0.67	0.15	0.64
6	0.57	4.63	0.64	4.23	*	2.70	*	2.40

**Table 5:** The average of SDNN in the wake–drowsiness state obtained from the analysis window.

Subject	SDNN by 60 s Window (ms)				SDNN by 300 s Window (ms)			
	ECG		Radar		ECG		Radar	
	Wake	Drowsiness	Wake	Drowsiness	Wake	Drowsiness	Wake	Drowsiness
1	63.96	80.74	92.10	293.10	55.32	86.20	80.17	276.33
2	45.67	48.24	282.66	284.73	53.80	67.25	230.69	260.10
3	48.91	64.03	157.02	296.04	45.79	67.02	160.97	242.32
4	25.38	32.32	222.52	195.04	34.85	37.40	240.13	207.84
5	51.38	60.55	125.68	201.96	52.05	66.48	122.68	203.76
6	37.60	50.80	114.41	94.15	51.81	54.98	149.19	103.00

**Table 6:** The average of RMSSD in the wake–drowsiness state obtained from the analysis window.

Subject	RMSSD by 60 s window (ms)				RMSSD by 300 s window (ms)			
	ECG		Radar		ECG		Radar	
	Wake	Drowsiness	Wake	Drowsiness	Wake	Drowsiness	Wake	Drowsiness
1	87.77	88.42	122.80	484.09	65.75	91.06	112.69	456.19
2	57.74	54.98	436.97	451.69	45.47	61.82	369.81	425.13
3	41.43	59.67	235.31	498.14	36.94	60.59	243.03	394.42
4	15.64	28.31	357.80	319.81	38.53	29.12	380.74	326.17
5	57.62	66.45	204.09	319.75	49.92	67.50	199.46	322.03
6	34.87	57.53	392.29	378.55	36.94	60.95	243.03	162.50
